# Shear-Thinning in Oligomer Melts—Molecular Origins and Applications

**DOI:** 10.3390/polym13162806

**Published:** 2021-08-20

**Authors:** Ranajay Datta, Leonid Yelash, Friederike Schmid, Florian Kummer, Martin Oberlack, Mária Lukáčová-Medvid’ová, Peter Virnau

**Affiliations:** 1 Institute of Physics, Johannes Gutenberg University, Staudingerweg 9, 55128 Mainz, Germany; rdatta@uni-mainz.de (R.D.); friederike.schmid@uni-mainz.de (F.S.); 2 Institute of Mathematics, Johannes Gutenberg University, Staudingerweg 9, 55128 Mainz, Germany; yelash@uni-mainz.de; 3 Department of Mechanical Engineering, Technische Universität Darmstadt, Otto-Berndt-Str. 2, 64287 Darmstadt, Germany; kummer@fdy.tu-darmstadt.de (F.K.); oberlack@fdy.tu-darmstadt.de (M.O.)

**Keywords:** shear flow, shear-thinning, semiflexible polymers, oligomers, heterogeneous multiscale methods, molecular dynamics, discontinuous Galerkin method, soft matter, non-Newtonian fluids

## Abstract

We investigate the molecular origin of shear-thinning in melts of flexible, semiflexible and rigid oligomers with coarse-grained simulations of a sheared melt. Entanglements, alignment, stretching and tumbling modes or suppression of the latter all contribute to understanding how macroscopic flow properties emerge from the molecular level. In particular, we identify the rise and decline of entanglements with increasing chain stiffness as the major cause for the non-monotonic behaviour of the viscosity in equilibrium and at low shear rates, even for rather small oligomeric systems. At higher shear rates, chains align and disentangle, contributing to shear-thinning. By performing simulations of single chains in shear flow, we identify which of these phenomena are of collective nature and arise through interchain interactions and which are already present in dilute systems. Building upon these microscopic simulations, we identify by means of the Irving–Kirkwood formula the corresponding macroscopic stress tensor for a non-Newtonian polymer fluid. Shear-thinning effects in oligomer melts are also demonstrated by macroscopic simulations of channel flows. The latter have been obtained by the discontinuous Galerkin method approximating macroscopic polymer flows. Our study confirms the influence of microscopic details in the molecular structure of short polymers such as chain flexibility on macroscopic polymer flows.

## 1. Introduction

Understanding the relation between viscosity and structure and its implications on macroscopic flow is of prime importance, particularly for semiflexible polymers, which are omnipresent in nature (DNA, actin filaments, microtubules), and synthetic polymers (polyelectrolytes, dendronised polymers). Technological applications are manifold and include, e.g., purifying DNA in microfluidic devices [1,2,3] or separation of polymers [4]. Computer simulations at multiple scales nowadays provide powerful tools to probe and understand fundamental structure-flow relations as well as their applications.

At the microscopic level, non-equilibrium molecular dynamics (NEMD) simulations have been applied for forty years [5,6,7,8,9,10,11,12,13,14,15,16] to study how macroscopic flow properties, such as shear-thinning, emerge from dynamics of microscopic structures. From the beginning, fundamental issues were approached from two sides. On the one hand, generic bead-spring models (similar to the ones applied in this work) were used to probe the general behaviour of polymer melts under shear, such as shear viscosity at zero shear rate [5], progressive alignment and elongation with shear and corresponding correlations with stress [7], the increase in viscosity when approaching the glass-transition in polymer melts and movement modes of individual chains [10]. On the other hand, research in this direction was also driven by modelling and comparing simulations with actual experiments involving specific polymers [6,8,9]. The underlying models tend to be more involved in these cases and include bending and even torsion terms absent in the early studies mentioned above. It therefore comes as a bit of a surprise that the specific influence of stiffness on shear-thinning in polymers has only recently come into the focus of attention. Particularly, Reference [13] provides a comprehensive study and already anticipates some of the effects also discussed in this work, while [14] focuses on the influence of chain stiffness on individual movement modes in single chain simulations in a MPCD solvent. We will argue, amongst others, that the emergence and decline of entanglements (e.g., as considered for melts of long flexible chains under shear in [17] and for polyethylene in [18,19]) may also play an important role for short oligomers in this context.

For complex multiscale systems, bridging over a large range of dynamically coupled scales is a challenging problem. In previous decades, this question has led to the development of new mathematical algorithms and hybrid multiscale methods. One possibility to build a multiscale algorithm relies on the Lagrangian–Eulerian decomposition, where the Lagrangian-type particles are embedded in the Eulerian description of fluid; see, e.g., [20,21,22]. Another approach is based on the domain decomposition. Hereby, a small accurate atomistic region is embedded into a coarser macroscopic model, see, e.g., [23]. Several hybrid models combining particle dynamics with the macroscopic continuum model can be found in the literature. In this context, we should mention, e.g., the hybrid heterogeneous multiscale methods [21,22,24,25,26,27,28], the seamless multiscale methods [29,30], the equation-free multiscale methods [31,32], the triple-decker atomistic-mesoscopic-continuum method [23], and the internal-flow multiscale method [33,34]. A nice overview of multiscale flow simulations using particles is presented in [35].

In this paper, we apply a hybrid multiscale method that couples atomistic details obtained by molecular dynamics with a continuum model approximated by the discontinuous Galerkin method. In order to extract mean flow field information from the molecular dynamics, averaging needs to be performed. Specifically, the required rheological information for the complex stress tensor is calculated by means of the Irving–Kirkwood formula [36]. Consequently, the averaged stress tensor is passed to the macroscopic continuum model. Thus, our method belongs to the class of hybrid particle-continuum methods under the statistical influence of microscale effects. We note in passing that the degree of scale separation of a physical system influences the sensitivity of the accuracy of a solution and the computational speed-up over a full molecular simulation [34].

The present paper is organised in the following way. First, we review and explore with NEMD simulations the molecular foundation of shear-thinning in low molecular weight polymers as a function of chain stiffness in the framework of a microscopic bead-spring model (Section 2 and Section 3). In particular, we will show how shear-thinning emerges from an intricate interplay of molecular alignment, stretching and tumbling modes [12,14,37]. The comparison of our high-density melt with simulations of single chains will also allow us to provide educated guesses for the density dependence of individual effects. In Section 4 and Section 5, we use microscopic data obtained from simulations in Section 3 as input for the study of various macroscopic channel flows using a hybrid multiscale method and investigate differences from Newtonian flow behaviour arising due to shear-thinning effects.

## 2. Microscopic Model and Simulation Techniques

For our microscopic model, we use standard bead-spring chains to represent the oligomers, as formulated by Kremer and Grest [38]. In this model, all beads interact with each other via a repulsive Weeks–Chandler–Andersen potential [39]:

(1) $$\begin{array}{cc} V_{WCA}\left(r\right) & =4\epsilon \left[{\left(\frac{\sigma }{r}\right)}^{12}-{\left(\frac{\sigma }{r}\right)}^{6}+\frac{1}{4}\right],\;r<2^{\frac{1}{6}}\sigma \\ \; & =0,\;r>2^{\frac{1}{6}}\sigma \end{array}$$

with σ=1 and ϵ=1. Adjacent beads are connected with an additional FENE interaction [40,41]:

(2) $$V_{FENE}=-\frac{1}{2}KR^{2}\ln\left[1-{\left(\frac{r}{R}\right)}^{2}\right]$$

with K=30 and R=1.5. Semiflexibility is implemented with a bending potential:

(3) $$V_{\theta }=\kappa \left(1+\cos\theta \right)$$

with θ being the angle between the three involved consecutive atoms and κ being the coefficient of stiffness. A cosine type bending potential such as Equation (3) originates from the well-known Kratky–Porod model [42,43,44] and is a common choice for modelling semiflexibility in polymers [45]. Note that even though our short flexible chains are essentially unentangled, Reference [46] suggests for a very similar model that the entanglement length drops steeply with increasing stiffness for semiflexible chains, implying that chains of length N=15 are already entangled for our intermediate range of stiffnesses (κ≈5).

Non-equilibrium molecular dynamics simulations of a sheared melt at density ρ=0.8 were performed using the LAMMPS simulation package [47]. System sizes were set to ${\left(15\sigma \right)}^{3}$ if not mentioned otherwise. Shear along the x-direction was introduced by superimposing a velocity gradient on thermal velocities using the SLLOD equations [48,49,50,51] and coupling the latter to the Nose–Hoover thermostat [50,52]. Temperature T = 1 was maintained throughout our simulations, and the Velocity Verlet algorithm was used to integrate the equations of motion. Note that LAMMPS implements a non-orthogonal simulation box with periodic boundary conditions that deforms continuously in accordance with the applied shear rate [53,54]—an approach that has been shown to be equivalent to the commonly used Lees–Edwards boundary conditions [51,55].

Shear viscosity $\eta (\dot{\gamma })$ was calculated using the relation

(4) $$\eta =\frac{\sigma_{xy}}{\dot{\gamma }},$$

where $\dot{\gamma }$ is the applied shear rate and $\sigma_{xy}$ is a non-diagonal component of the stress tensor as determined by the Irving–Kirkwood formula [36,56]:

(5) $$\sigma_{xy}=-\frac{1}{V}\left[\sum_{i}^{N}\left(m_{i}v_{i,x}v_{i,y}\right)+\sum_{i}^{N}\sum_{j>i}^{N}\left(r_{ij,x}f_{ij,y}\right)\right].$$

Here, $m_{i}$ is the mass of the *i*th particle, $v_{i}$ the peculiar velocity of the *i*th particle, and $r_{\mathrm{ij}}$ and $f_{\mathrm{ij}}$ are the distance and force vectors between the *i*th and the *j*th particle, respectively. For comparison, we have also calculated shear viscosity via the Green–Kubo relation:

(6) $$\eta_{GK}=\frac{V}{k_{B}T}\int_{0}^{\infty }\langle \sigma_{xy}\left(t\right)\sigma_{xy}\left(0\right)\rangle dt.$$

where *V* is the volume of the system, and $k_{B}$ is the Boltzmann constant. $\eta_{GK}$ is measured under equilibrium conditions ($\dot{\gamma }=0$) and serves as a reference value for $\eta (\dot{\gamma }\to 0)$.

Note that forces arising from the thermostat and its coupling to the SLLOD conditions are not explicitly considered in Equation (5) and may potentially result in a small systematic error. For a detailed discussion of this effect in the context of dissipative thermostats, the reader is referred to Reference [57].

In addition, we have carried out Brownian dynamics simulations of single chains (same potentials as above) in an external shear flow profile. The equation of motion of monomers *i* is given by

(7) $$\dot{r_{i}}=\frac{1}{\zeta }f_{i}+\dot{\gamma }z_{i}+\xi_{i}\left(t\right),$$

where ζ is the monomeric friction, $f_{i}$ the intermolecular force acting on *i*, and $\xi_{i,\alpha }\left(t\right)$ an uncorrelated Gaussian white noise with mean zero obeying the fluctuation–dissipation theorem, i.e., $\langle \xi_{i\alpha }\left(t\right)\xi_{i\beta }\left(t^{\prime }\right)\rangle =\left(2k_{B}T/\zeta \right)\;\delta_{ij}\delta_{\alpha \beta }\delta \left(t-t^{\prime }\right)$. In the simulations, we used an Euler forward algorithm with a time step $\Delta t=10^{-4}\zeta \sigma^{2}/\epsilon$. The time scales of the two models were adjusted by mapping the Rouse times of fully flexible chains (κ=0) at equilibrium ($\dot{\gamma }=0$). To determine the Rouse time, we determined the autocorrelation function of the squared end-to-end distance,

(8) $$C_{R_{ee}}\left(t\right)=\frac{\langle R_{ee}^{2}\left(t\right)R_{ee}^{2}\left(0\right)\rangle -{\langle R_{ee}^{2}\rangle }^{2}}{\langle R_{ee}^{4}\rangle -{\langle R_{ee}^{2}\rangle }^{2}}.$$

For ideal Rouse chains, it can be calculated analytically, giving

(9) $$C_{R_{ee}}\left(t\right)={\left(\frac{8}{\pi^{2}}\sum_{p\mathrm{odd}}\frac{1}{p^{2}}\;e^{-p^{2}{\left(t/\tau_{R}\right)}^{2}}\right)}^{2},$$

where *p* sums over the Rouse modes of the chain. At late times, the behaviour is dominated by the first Rouse mode with p=1. We thus fitted the late time behaviour of $\sqrt{C_{R_{ee}}\left(t\right)}$ to the function $Ae^{-t/\tau_{R}}$ for chains of length N=15 in a melt and for the corresponding single Brownian chains. The fit parameters for the prefactor *A* were in rough agreement with the theoretical value $8/\pi^{2}=0.81$ in both cases (A=0.87 for melt chains, and A=0.90 for single oligomers). The fitted Rouse time of melt chains was $\tau_{R}=\left(85.4\pm 0.1\right)\sqrt{m\sigma^{2}/\epsilon }$, and that of single oligomers was $\tau_{R}=\left(8.45\pm 0.02\right)\zeta \sigma^{2}/\epsilon$. Hence, the time scales match when choosing $\zeta =10.1\sqrt{m\epsilon /\sigma^{2}}$. We have also carried out a more intricate mapping (discussed in Appendix A), which matches Rouse times for each value of κ, but does not change our results qualitatively.

## 3. Shear-Thinning in Oligomer Melts—A Molecular Analysis

In the following section, we would like to investigate and review the molecular foundation of shear-thinning in low molecular weight polymer melts. We will show and highlight that macroscopic flow properties of polymers are governed by an intricate interplay of stretching, alignment and tumbling of individual molecules as well as collective modes at the molecular level.

**Figure 1:**
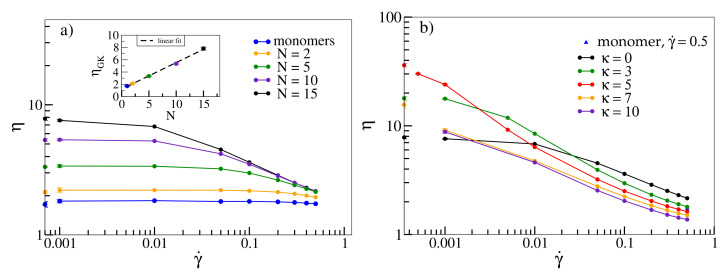
(**a**) Viscosity η as a function of shear rate $\dot{\gamma }$ for a melt of flexible oligomer chains (κ=0) with N = 1, 2, 5, 10 and 15 beads per chain. Corresponding shear viscosities according to the Green–Kubo relation $\eta_{GK}$ are shown on the *y*-axis and displayed as a function of *N* in the inset. Density ρ=0.8 and box dimensions are $10\times 10\times 10\sigma^{3}$ for N = 1, 2, 5 and 10 and $15\times 15\times 15\sigma^{3}$ for N = 15. (**b**) $\eta (\dot{\gamma })$ for a melt with N = 15 and ρ=0.8 and varying stiffnesses. $\eta_{GK}$ for κ=0,3,5,7 and 10 are displayed on the *y*-axis. The viscosity for monomers at $\dot{\gamma }$ = 0.5 (blue triangle) is also shown for reference. If not displayed explicitly, errors are smaller than symbol sizes. All lines serve as guides to the eye.

Figure 1a displays viscosity η as a function of shear rate $\dot{\gamma }$ for flexible oligomers with different chain lengths *N*. Flexible oligomers exhibit shear-thinning [10], i.e., decrease of viscosity with increasing shear rate. For consistency, we also compare viscosities as derived from Equation (5) with those obtained from the Green–Kubo relation, Equation (6) (points on the *y*-axis). The latter agree with the values for $\dot{\gamma }=0.001$ within the error bars. The overall shape of $\eta (\dot{\gamma })$, namely a plateau at low shear rates followed by a shear-thinning regime, which becomes more pronounced with increasing molecular weight, has also been observed for various polymers experimentally [9,58,59]. The inset shows that $\eta_{GK}$ increases linearly with *N* for small chain lengths in agreement with simulations from [5].

In Figure 1b, we investigate the dependence of viscosity on shear rate as a function of stiffness for an oligomer melt with chain length N=15. While for large shear rates, viscosity decreases with increasing stiffness and intriguingly even drops below the value determined for monomers (for κ>3), η(κ) becomes non-monotonic for low shear rates. For $\dot{\gamma }=0.001$, flexible chains with κ=0 have the lowest viscosity, while viscosity increases for semiflexible chains and drops down again for rigid chains, an effect described in [13] for a similar model. A similar non-monotonic behaviour is exhibited by $\eta_{GK}$ (shown on the margins of Figure 1b as a function of κ). While the rise of viscosity can already be explained with the emergence of entanglements for intermediate stiffnesses, at the end of this section we will associate the following decline with a collective alignment of chains (and associated disentanglements), which are amplified by shear (Figure 4).

**Figure 2:**
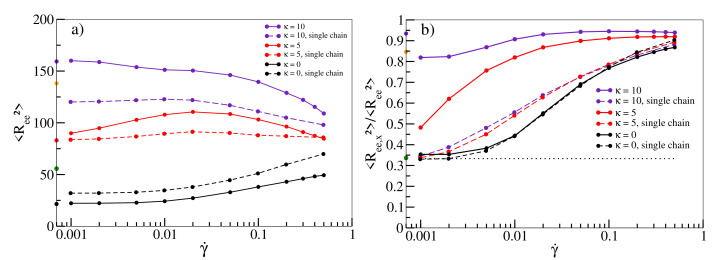
(**a**) $\langle R_{ee}^{2}\rangle$ as a function of shear rate $\dot{\gamma }$ for stiffnesses κ=0, κ=5 and κ=10 at density ρ=0.8. (**b**) Ratio of the x-component $\langle R_{ee,x}^{2}\rangle$ and $\langle R_{ee}^{2}\rangle$ as a function of $\dot{\gamma }$ for κ=0, κ=5 and κ=10. The dotted line at the ratio of 1/3 marks the value for an unsheared melt. Results for a single chain in shear flow are shown as dashed lines (with points) in both figures. Values on the *y*-axis (in (**a**,**b**), colour scheme such as in Figure 1b) correspond to equilibrium simulations without shear. As there is no preferred orientation in the bulk, the value for the ratio refers to the largest component. For κ≤5, there is no preferred orientation in the bulk. All lines serve as guides to the eye.

In Figure 2a, we quantify the stretching of individual chains with shear. The size of a flexible chain as measured by the mean square end-to-end distance $\langle R_{ee}^{2}\rangle$ increases continuously as a function of shear rate. For κ=5, the average size only increases slightly towards moderate shear rates before decreasing again at high rates similar to [14], ruling out stretching as a main driving force for shear-thinning in this regime for melts of semiflexible chains. Figure 2b visualises the alignment of chains along the shear direction by plotting the ratio of the x-component to the total $\langle R_{ee}^{2}\rangle$. Without shear, each component contributes equally, yielding a ratio of 1/3 (dotted line in Figure 2b). While this holds for flexible chains at low shear rates, deviations become more pronounced for shear rates exceeding 0.01. This is also roughly the rate at which noticeable deviations from $\eta_{GK}$ start to occur in Figure 1a and shear-thinning sets in. This behaviour becomes even more pronounced for semiflexible chains. At $\dot{\gamma }=0.001$, chains are already partially aligned, and the viscosity in Figure 1b already deviates significantly from the value obtained from the Green–Kubo relation. Shear-thinning sets in at even lower shear rates and is reinforced with progressive alignment of chains. This relation provides a clear indication that chain alignment is strongly correlated with the occurrence of shear-thinning in agreement with previous observations [13,14]. Note that for κ>5, chains are already stretched and aligned in equilibrium simulations without shear (values on *y*-axis of Figure 2a,b) indicating the emergence of nematic behaviour in agreement with previous observations in a similar model [46,60]. For κ=10, there is even an initial drop from the bulk ratio once shear sets in. Interestingly, stretching and alignment of chains can already be observed qualitatively in simulations of single chains in shear flow (dashed lines in Figure 2a,b), indicating that these phenomena should in principle be observable for melts of all densities. However, while the alignment of flexible chains is well-reproduced, the increase is less pronounced for higher stiffnesses, indicating that collective alignment contributions due to stiffness are not captured by single chain simulations.

In the following, we turn to movement modes of individual chains.

**Figure 3:**
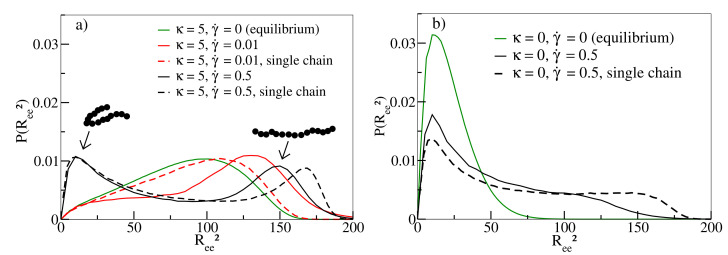
(**a**) Probability distributions P($R_{ee}^{2}$) for stiffness κ = 5 at shear rates $\dot{\gamma }=0,0.01$ and 0.5. The two peaks of the distribution for $\dot{\gamma }=0.5$ correspond to U-shaped configurations and stretched configuration of individual oligomers, respectively, as indicated by typical snapshots. (**b**) P($R_{ee}^{2}$) for stiffness κ = 0 and shear rate $\dot{\gamma }=0$ and 0.5. Results for a single chain in shear flow are shown as dashed lines in both figures.

Figure 3 shows the distribution of $R_{ee}^{2}$ of individual chains in melts. While equilibrium simulations ($\dot{\gamma }=0$) have a broad distribution of end-to-end distances for κ=5 (solid green line in Figure 3a), conformations develop a preference for stretched chains at moderate shear rates ($\dot{\gamma }=0.01$, solid red line). At about this rate, $\langle R_{ee}^{2}\rangle$ displays a maximum in Figure 2a. For the highest shear rate $\dot{\gamma }=0.5$ (solid black line), U-shaped tumbling conformations coexist with stretched conformations explaining the decrease of $\langle R_{ee}^{2}\rangle$ in Figure 2a for $\dot{\gamma }>0.02$[14]. For flexible chains, compact conformations dominate the behaviour at small and large shear rates (Figure 3b), as already noted in [10], even though the latter also exhibit some degree of stretched conformations. This also explains why $\langle R_{ee}^{2}\rangle$ is significantly smaller in comparison to semiflexible chains (Figure 2a). It is worth noting that the occurrence of compact conformations does not impede the continuous alignment of chains along the shear direction with increasing shear rate, as demonstrated in Figure 2b. The intricate interplay between stretching and tumbling as a function of shear rate and stiffness can also be observed for our single chain simulations (as noted for flexible chains already in [10] and studied in [11]), indicating that these movement modes are not a collective phenomenon and should occur in melts of all densities [15].

**Figure 4:**
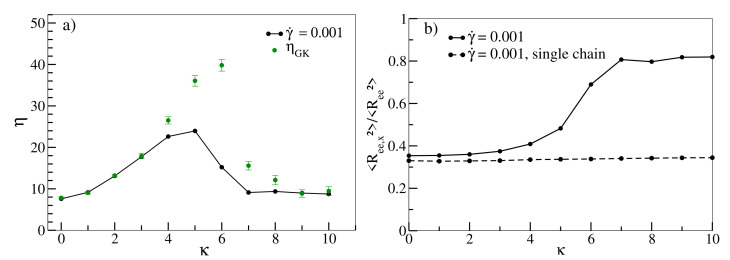
(**a**) Viscosity η as a function of stiffness κ for shear rate $\dot{\gamma }$ = 0.001. Shear viscosity at zero shear rate $\eta_{GK}$ are shown as green dots. (**b**) $\langle R_{ee,x}^{2}\rangle /\langle R_{ee}^{2}\rangle$ as a function of κ at $\dot{\gamma }=0.001$ for melt and single chain simulations (dashed lines). All lines serve as guides to the eye.

**Figure A2:**
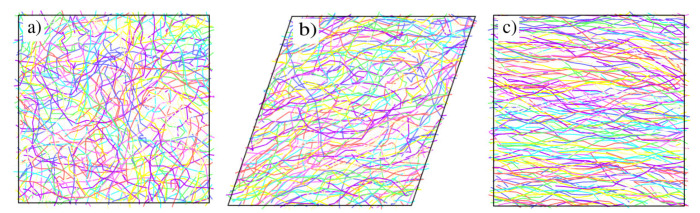
Snapshots of melts corresponding to (**a**) κ=6 in equilibrium, (**b**) κ=6 at $\dot{\gamma }=0.001$ (**c**) κ=10 in equilibrium.

In Figure 4, we finally investigate the non-monotonous behaviour of the Green–Kubo viscosity $\eta_{GK}$ and viscosity at low shear rates ($\dot{\gamma }=0.001$) as functions of κ. $\eta_{GK}$ increases with increasing chain stiffness, reaches a maximum at about κ=6 and undergoes a sharp decrease after that. Furthermore, $\eta (\dot{\gamma }=0.001)$ increases up to κ=5 and decreases subsequently. Intriguingly, $\eta_{GK}$ matches with $\eta (\dot{\gamma }=0.001)$ up to κ=3, but, between κ=4 and κ=6, $\eta_{GK}$ is significantly larger than $\eta (\dot{\gamma }=0.001)$. As already pointed out, Reference [46] estimates that the entanglement length decreases with increasing persistence length, $l_{p}$, to a point at which the entanglement length becomes smaller than the chain size. It estimates that at $l_{p}=1.5,3,5$, the entanglement lengths are approximately equal to 15, 8 and 6, respectively. Reference [61] estimates that the numerical values of $l_{p}$ are quite close to the numerical values of κ. For example, κ=3 and 5 correspond to $l_{p}\approx$ 2.5 and ≈5, respectively. Therefore, chains become entangled, and this effect increases with increasing κ. It should be noted, however, that for both Reference [46] and [61], the number density was 0.85, which is a bit higher than the number density of our system (0.8). Bond and angular potentials forms also differ slightly in [46]. Incidentally, Reference [62] also obtains an analytic expression that estimates entanglement length as a function of chain stiffness for isotropic polymer chains. Unaligned and entangled chains impede collective motion under equilibrium conditions, and as a result, $\eta_{GK}\left(\kappa \right)$ increases up to κ=6. Following κ=6, however, there is a sharp drop in $\eta_{GK}$. This is consistent with our observation from Figure 2b that for κ=7 and κ=10, chains are already stretched and aligned under equilibrium conditions, indicating that our system indeed undergoes an isotropic-nematic transition following κ=6. Entanglements decrease as chains align and conformations become more susceptible to the applied shear, resulting in a decrease in $\eta_{GK}$. $\eta (\dot{\gamma }=0.001)$ as a function of κ also exhibits a similar non-monotonous behaviour. While the initial increase can also be attributed to progressive entanglements, for κ≥4, the applied shear already begins to align the chains towards the shear direction, counteracting entanglement effects, as is evident in Figure 4b. As a result, $\eta (\dot{\gamma }=0.001)$ are lower than the corresponding $\eta_{GK}$ values. For κ≥6, chains are more strongly aligned along the shear direction, thus $\eta (\dot{\gamma }=0.001)$ as a function of κ decreases beyond κ=5 [13]. This behaviour is, in contrast to stretching and alignment with increasing shear rate, a collective phenomenon that cannot be observed in corresponding simulations of single chains and should therefore vanish gradually at smaller densities. Our finding that rheological properties in rather short oligomeric systems are dominated by the emergence and decline of entanglements may come as a surprise. However, it should be noted that some prior studies have also associated shear-thinning with decreasing entanglements, albeit for much longer chains. Reference [18] shows that in a linear polyethylene melt ($C_{400}H_{802}$) system comprising polymers with a finite stiffness, the number of entanglement strands per chain decrease with increasing shear rate. Reference [19] argues along similar lines for a melt comprising of even longer linear polyethylene chains ($C_{700}H_{1402}$), and Reference [17] shows a similar decrease in entanglements with increasing shear rate for flexible polymer chains (N=200 and 400). In Appendix B, Figure A2 displays various configuration snapshots of oligomer melts, which further visualise the interplay between disentanglement, alignment and shear.

## 4. Hybrid Multiscale Method

After studying the molecular origin of non-Newtonian behaviour of short polymer melts, we now turn our attention to macroscopic simulations combining them with the results of molecular dynamics.

The motion of an incompressible fluid flow at the macroscopic level is governed by the continuity and the momentum equations

(10a) $$\begin{array}{cccc} & \nabla \cdot u=0, & \; & \mathrm{in}\;\Omega \times [0,T] \end{array}$$

(10b) $$\begin{array}{cccc} & \frac{\partial u}{\partial t}+u\cdot \nabla u=\nabla \cdot \sigma +g,\; & & \mathrm{in}\;\Omega \times [0,T] \end{array}$$

(10c) $$\begin{array}{cccc} & u=u_{D}, & & \mathrm{on}\;\partial \Omega_{D} \end{array}$$

(10d) $$\begin{array}{cccc} & \sigma \cdot n=0, & & \mathrm{on}\;\partial \Omega_{N} \end{array}$$

(10e) $$\begin{array}{cccc} & u\left(t=0\right)=u^{\left(0\right)} & & \mathrm{in}\;\Omega , \end{array}$$

where u is the velocity vector, σ the Cauchy stress tensor, and g an external body force. The boundary of the computational domain Ω consists of the Dirichlet, Neumann and periodic boundary, i.e., $\partial \Omega =\partial \Omega_{D}\cup \partial \Omega_{N}\cup \partial \Omega_{P}$.

The Cauchy stress tensor can be split into two parts σ=−pI+τ with *p* being an isotropic hydrostatic pressure and τ a viscous stress tensor. For the Navier–Stokes equations, we have $\tau =\eta (\nabla u+\nabla u^{T})$ with η being a constant viscosity. This relation is more complex when non-Newtonian polymer fluids are considered.

In this work, we apply the hybrid multiscale method that couples the molecular dynamics simulations with the macroscopic model (10a–e). As explained in Section 2, the macroscopic stress tensor can be derived from the Irving–Kirkwood formula (5).

Our extensive molecular dynamics simulations imply that the stress tensor can actually be expressed in the following simple way

(11) $$\tau =\eta \left(\dot{\gamma }\right)\left(\nabla u+\nabla u^{T}\right).$$

Finally, the viscosity–shear rate dependence leads to a well-known Carreau–Yasuda rheological model [63]

(12) $$\eta \left(\dot{\gamma }\right)=\eta_{\infty }+\left(\eta_{0}-\eta_{\infty }\right){\left(1+{\left(a_{2}\dot{\gamma }\right)}^{a_{3}}\right)}^{a_{1}}$$

with the following coefficients: for flexible polymers (stiffness κ=0) $\eta_{0}=7.76$, $\eta_{\infty }=1.08441$, $a_{1}=-0.425387$, $a_{2}=54.3905$, $a_{3}=1.28991$; and for semiflexible polymers with κ = 5 $\eta_{0}=36.052$, $\eta_{\infty }=1.09319$, $a_{1}=-0.214969$, $a_{2}=2143.96$, $a_{3}=2.78713$.

We note in passing that for a particular situation considered in this paper, our hybrid multiscale method can be seen as a parameter passing sequential coupling multiscale method, see, e.g., ref. [29] for a detailed description of the concurrent and sequential coupling strategies.

**Figure 5:**
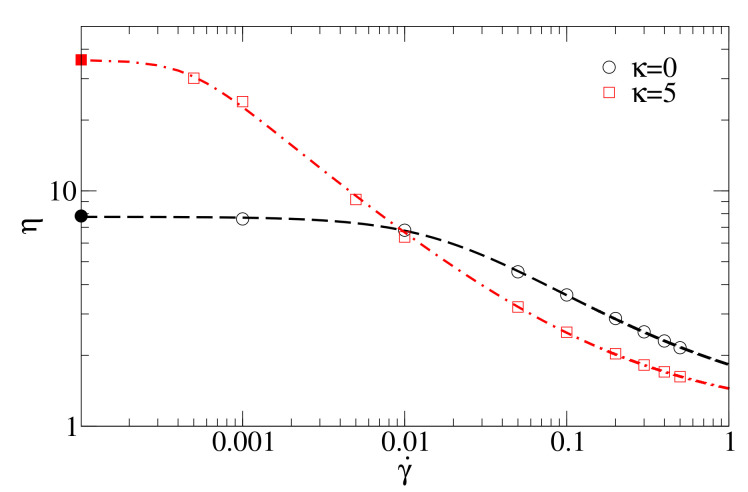
Shear viscosity of flexible oligomer chains and oligomer chains with stiffness κ=5 as obtained by non-equilibrium molecular dynamics (open symbols) and fitting by the Carreau–Yasuda rheological fluid model Equation (12) (dashed curves, compare with Figure 1b). Bold symbols on the *y*-axis represent viscosity values at $\dot{\gamma }=0$ obtained from equilibrium molecular dynamics simulations.

The oligomer chain length in both cases is N=15. Figure 5 compares the MD data and the fitting with the Carreau–Yasuda model.

Our next goal is to calculate the shear-dependent viscosity $\eta (\dot{\gamma }).$ In what follows, we consider for simplicity the situation of two-dimensional shear flows and use the notation u=(u,v). Applying (12), we need the value of the shear rate $\dot{\gamma }$ of the polymer flow. It can be obtained from the strain-rate tensor

(13) $$S=\frac{\nabla u+\nabla u^{T}}{2}=\left(\begin{array}{cc} \frac{\partial u}{\partial x} & \frac{\left(\frac{\partial u}{\partial y}+\frac{\partial v}{\partial x}\right)}{2}\\ \frac{\left(\frac{\partial u}{\partial y}+\frac{\partial v}{\partial x}\right)}{2} & \frac{\partial v}{\partial y} \end{array}\right)$$

by rotating it with respect to the streamlines to the anti-diagonal matrix

(14) $$S^{\prime }=\Theta S\Theta^{T}=\left(\begin{array}{cc} 0 & \dot{\gamma }/2\\ \dot{\gamma }/2 & 0 \end{array}\right)\;$$

by an angle θ, which for incompressible flows is $\theta =\frac{1}{2}\tan^{-1}\left(-\frac{S_{xx}}{S_{xy}}\right)$. Here, $S_{ij}$ are components of the strain-rate tensor S and Θ the rotation matrix [64,65]. The new strain-rate tensor $S^{\prime }$ corresponds to a pure-shear deformation (i.e., in absence of normal stresses). The shear rate can therefore be calculated from the components of the original strain-rate tensor S and the angle θ

(15) $$\dot{\gamma }/2=S_{xy}\cos\left(2\theta \right)-S_{xx}\sin\left(2\theta \right).$$

The stress tensor in the shear flow can be calculated in the normal-stress free basis and transformed back to the original basis according to (11)

(16) $$\tau =\Theta^{T}\left(2\eta \left(\dot{\gamma }\right)S^{\prime }\right)\Theta .$$

As shown in (16), we consider in the present work shear dependent flows, where the stress tensor or, more precisely, the viscosity are nonlinear functions of the local shear rate. In order to consider more general flow conditions, such as the extensional flow, rigid rotation and mixed flows, one needs to take into account not only the shear rate dependence but a complete decomposition of a three-dimensional symmetric tensor (stress tensor) into a six-dimensional basis. In such a way, not only the viscosity but also additional response coefficients will be computed from microscopic simulations in order to determine the local stress tensor. We refer the reader to our recent work [66] where complex flows in general geometries were studied, see also [67].

We proceed by describing a numerical method applied to (10a–e). For *time integration*, we apply the implicit BDF2 scheme, which leads to the following system

(17a) 32u(n+1)+Δtu(n+1)·∇u(n+1)+∇p(n+1)−∇·τ(n+1)−g(n+1)mm=2u(n)−12u(n−1)inΩ

(17b) $$\begin{array}{ccccc} & \; & & \nabla \cdot u^{\left(n+1\right)}=0\;\;\mathrm{in}\;\Omega . & \end{array}$$

Discretisation in space is realised by the discontinuous Galerkin (dG) method [68,69,70]. Domain Ω is discretised by a quadrilateral mesh $T_{h}$ with a meshsize *h*. Mesh faces $F_{h}$ can be either the inner interfaces between adjacent elements $F_{h}^{i}$ or boundary faces $F_{h}^{b}$. The face normal $n_{F}$ points from an arbitrarily chosen (but fixed) element $T_{1}$ towards $T_{2}$. The face normal points outward of the domain Ω on boundary faces.

We consider broken Sobolev spaces $V_{h},\;Q_{h}$ that consist of piecewise quadratic and piecewise linear polynomials, respectively,

(18a) $$\begin{array}{cc} & V_{h}:=\{v\in L^{2}\left(\Omega \right)|\;\mathrm{for}\;\mathrm{all}\;T\in T_{h},v{|}_{T}\;\mathrm{is}\;a\;\mathrm{quadratic}\;\mathrm{polynomial}\} \end{array}$$

(18b) $$\begin{array}{cc} & Q_{h}:=\{q\in L^{2}\left(\Omega \right)|\;\mathrm{for}\;\mathrm{all}\;T\in T_{h},q{|}_{T}\;\mathrm{is}\;a\;\mathrm{linear}\;\mathrm{polynomial}\}. \end{array}$$

The usual average and jump operators of a scalar-valued function $f_{h}$ on interfaces between adjacent elements $T_{1}$ and $T_{2}$ are defined as

(19) $$\left\{\;\left\{f_{h}\right\}\;\right\}=\frac{1}{2}{\left(f\right|}_{T_{1}}{+f|}_{T_{2}}),\;\left[\;\left[f_{h}\right]\;\right]=f{{|}_{T_{1}}-f|}_{T_{2}}.$$

Vector-valued functions are treated componentwisely. For boundary faces, we set $\left\{\;\left\{f_{h}\right\}\;\right\}=\left[\;\left[f_{h}\right]\;\right]{=f|}_{T}$, when not mentioned otherwise.

The Discontinuous Galerkin (dG) method for an oligomer fluid flow (10a–e) is formulated as follows. Given the initial data $u_{h}^{\left(0\right)}\in V_{h}$, we look for a sequence of numerical solutions $u_{h}^{\left(n+1\right)}\in V_{h},$ $p_{h}^{\left(n+1\right)}\in Q_{h}$ for $n=1,\cdots ,N_{T}-1$ such that

(20a) $$\begin{array}{cc} \; & \left(\frac{3}{2}u_{h}^{\left(n+1\right)},\varphi_{h}\right)+\Delta t\;\left(\;c\left(u_{h}^{\left(n+1\right)},u_{h}^{\left(n+1\right)},\varphi_{h}\right)-b\left(p_{h}^{\left(n+1\right)},\varphi_{h}\right)+a\left(\eta \left({\dot{\gamma_{h}}}^{\left(n+1\right)}\right),u_{h}^{\left(n+1\right)},\varphi_{h}\right)\right)\\ \; & =\Delta t\;\left(g_{h}^{\left(n+1\right)},\varphi_{h}\right)+\left(2u_{h}^{\left(n\right)}-\frac{1}{2}u_{h}^{\left(n-1\right)},\varphi_{h}\right) \end{array}$$

(20b) $$\begin{array}{cc} \; & b\left(q_{h},u_{h}^{\left(n+1\right)}\right)=0\;\;\mathrm{for}\;\mathrm{any}\;\varphi_{h}\in V_{h},\;q_{h}\in Q_{h}. \end{array}$$

The numerical shear rate ${\dot{\gamma_{h}}}^{\left(n+1\right)}$ is computed locally, i.e., in each quadrature point from the broken gradients $\nabla u_{h}^{\left(n+1\right)}$. Hereby, we compute the first approximation $u_{h}^{\left(1\right)}$, e.g., by the Euler implicit method. The $L^{2}\left(\Omega \right)$ scalar product is denoted by (·,·). In what follows, we define the discrete forms *a*, *b*, and c.

The *convective term* is rewritten in the conservative form and the interface integrals are approximated by means of the Lax–Friedrichs numerical flux

(21) $$c\left(u_{h},w_{h},\varphi_{h}\right)=-\int_{\Omega }\left(u_{h}⊗w_{h}\right):\nabla \varphi_{h}+\int_{F_{h}}\left(\left\{\;\left\{u_{h}⊗w_{h}\right\}\;\right\}\cdot n_{F}+\frac{1}{2}\Lambda \left[\;\left[u_{h}\right]\;\right]\;\right)\cdot \left[\;\left[\varphi_{h}\right]\;\right]$$

where $\Lambda =\max(\lambda {|}_{T_{1}},\lambda {|}_{T_{2}})$, and λ is the absolute eigenvalue of the Jacobian matrix $\left(\partial [\left(u⊗w\right)\cdot n_{F}]/\partial u\right){|}_{\bar{u},\bar{w}}$. The average and jump operators on the Dirichlet boundaries are defined as

(22a) $$\begin{array}{ccc} & \left\{\;\left\{u_{h}⊗w_{h}\right\}\;\right\}=\frac{1}{2}\left(\left(u_{h}⊗w_{h}\right){|}_{T}+\left(u_{D}⊗w_{D}\right)\right),\;\left[\;\left[u_{h}\right]\;\right]=(u_{h}{|}_{T}-u_{D})\; & F\subset \partial \Omega_{D} \end{array}$$

(22b) $$\begin{array}{ccc} & \left\{\;\left\{u_{h}⊗w_{h}\right\}\;\right\}=\left(u_{h}⊗w_{h}\right){{|}_{T},\;\left[\;\left[u_{h}\right]\;\right]=u_{h}|}_{T}\; & F\subset \partial \Omega_{N} \end{array}$$

(22c) $$\begin{array}{ccc} & \left[\;\left[\varphi_{h}\right]\;\right]=\varphi_{h}{|}_{T}\; & F\subset \partial \Omega_{D}\cup \partial \Omega_{N}. \end{array}$$

The divergence of the velocity $u_{h}$ as well as the discrete gradient of the pressure $p_{h}$ are both approximated using the form

(23) $$b\left(r_{h},\phi_{h}\right)=-\int_{\Omega }\phi_{h}\cdot \nabla r_{h}+\int_{F_{h}\backslash \partial \Omega_{N}}\left\{\;\left\{\phi_{h}\right\}\;\right\}\cdot n_{F}\left[\;\left[r_{h}\right]\;\right].$$

To discretize the viscous, terms we employ an almost-standard symmetric interior penalty method (SIP), first introduced in [71] and extensively analysed in [72]

(24) $$\begin{array}{c} a\left({\dot{\gamma }}_{h},u_{h},\varphi_{h}\right)=\int_{\Omega }\eta \left({\dot{\gamma }}_{h}\right)\left(\nabla u_{h}+\nabla u_{h}^{T}\right):\nabla \varphi_{h}+\int_{F_{h}\backslash \partial \Omega_{N}}\mu_{P}\eta \left({\dot{\gamma }}_{h}\right)\left[\;\left[u_{h}\right]\;\right]\cdot \left[\;\left[\varphi_{h}\right]\;\right]\\ -\int_{F_{h}\backslash \partial \Omega_{N}}\left(\left\{\;\left\{\eta \left({\dot{\gamma }}_{h}\right)\left(\nabla u_{h}+\nabla u_{h}^{T}\right)\right\}\;\right\}n_{F}\right)\cdot \left[\;\left[\varphi_{h}\right]\;\right]+\left(\left\{\;\left\{\eta \left({\dot{\gamma }}_{h}\right)\left(\nabla \varphi_{h}+\nabla \varphi_{h}^{T}\right)\right\}\;\right\}n_{F}\right)\cdot \left[\;\left[u_{h}\right]\;\right]. \end{array}$$

On the Dirichlet boundaries $F\subset \partial \Omega_{D}$, we set

(25) $$\left\{\;\left\{\ldots \right\}\;\right\}=\left(\ldots \right){{|}_{T},\;\left[\;\left[u_{h}\right]\;\right]=\left(u_{h}{|}_{T}-u_{D}\right),\;\left[\;\left[\varphi_{h}\right]\;\right]=\varphi_{h}|}_{T}.$$

Coefficient $\mu_{P}$ is a penalty parameter that we choose for a quadraliteral mesh in the following way [73]

(26a) $$\begin{array}{cc} \; & \mu_{P}=\alpha_{P}c=\left\{\begin{array}{cc} \alpha_{P}{\max(c|}_{T_{1}},c{|}_{T_{2}}) & F\in F_{h}^{i}\\ \alpha_{P}{\;c|}_{T}, & F\in F_{h}^{b} \end{array}\right. \end{array}$$

(26b) $$\begin{array}{cc} \; & {c|}_{T}=3^{2}\frac{A\left(\partial T\backslash F_{h}^{b}\right)/2+A\left(\partial T\cap F_{h}^{b}\right)}{V(T)}, \end{array}$$

where $\alpha_{P}\geq 1$ is a user-defined small coefficient. Area and volume are denoted by *A* and *V*, respectively. We conclude this section by mentioning that the resulting nonlinear system is approximated by the Newton method employing the Dogleg-globalisation and a direct sparse solver for the linearised system; see [74].

## 5. Two Channel Flows of a Non-Newtonian Oligomer Fluid

In this section, we illustrate the consequences of shear-thinning, whose microscopic origins have been discussed in previous sections, on two examples of macroscopic channel flows. Numerical simulations have been realised within BoSSS code, for more details see also [20,68,69,74,75].

In the first test, the so-called Poiseuille flow [76] is simulated. Here, an oligomer melt flows through a narrow channel of length ℓ=1, driven by a pressure difference between the outlet and inlet of the channel. The intensity of the flow is controlled by the pressure parameter related to the external pressure gradient $P_{x}=\left(P_{\mathrm{in}}-P_{\mathrm{out}}\right)/\ell =-\partial p/\partial x$. Since the viscosity is a function of the shear rate, we compute the Reynolds number using the averaged viscosity $\bar{\eta }=\left(\eta_{0}+\eta_{\infty }\right)/2,$ i.e.,

(27) $$Re=\frac{U\;L}{\bar{\eta }},$$

where *U* is the characteristic velocity (maximal inflow velocity) and *L* the characteristic length, i.e., the channel diameter L=1. In order to also take into account the effects of asymptotic viscosity values, we define $Re_{0}=UL/\eta_{0}$, $Re_{\infty }=UL/\eta_{\infty }$ and introduce them in Table 1.

**Figure 6:**
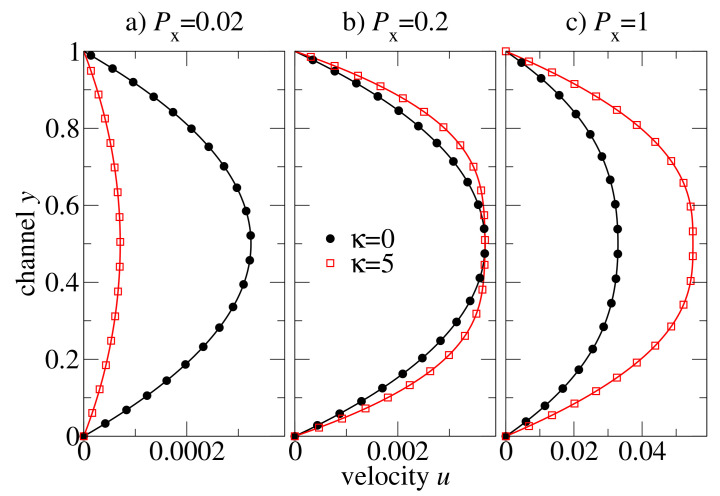
Steady-state velocity profiles of the pressure-driven channel flow of oligomer melts consisting of either flexible or semiflexible chains with stiffness κ=5. Solutions are computed by a hybrid MD-dG method (20b) for various external pressure difference parameters $P_{x}$.

For the macroscopic model of our hybrid multiscale method, we have used a grid with 128×1024 mesh cells, i.e., the mesh parameter h=(1/128,1/1024). Numerical simulations on a coarser grid confirm that the steady-state results presented here are independent on the mesh resolution. Figure 6 shows the steady-state velocity profiles across the channel for flexible (κ=0) and semiflexible (κ=5) polymer chains of the length N=15. The external pressure drop is chosen at values of $P_{x}=\left\{0.02,0.2,1\right\}$ in order to study the influence of the different regimes of the shear rate-dependent viscosity on an oligomer flow. At low-pressure drop ($P_{x}=0.02$ in Figure 6a), the flow is very slow and the shear rates are low, too. The viscosity of both polymeric systems is at the Newtonian plateau (cf. Figure 5). The viscosity of flexible chains is lower than that of the semiflexible chains (ca. 8 vs. 36), and therefore, the velocity of the flexible chains is higher than that of the semiflexible chain melt. At moderate pressure drop ($P_{x}=0.2$ in Figure 6b), the viscosity of the flexible chains is still nearly a constant (the Newtonian regime), whereas the semiflexible chain melt is already in the shear-thinning regime. By coincidence, the amplitude of the two profiles at the centre of the channel is approximately the same, and one can easily recognise that the shapes of the velocity profiles are very different: the semiflexible chains exhibit a broader distribution (Figure 6b). Further increase in the pressure drop leads to an inverse situation: the melt of semiflexible chain flows faster through the channel than the flexible chains melt (cf. $P_{x}=1$ in Figure 6c).

**Figure 7:**
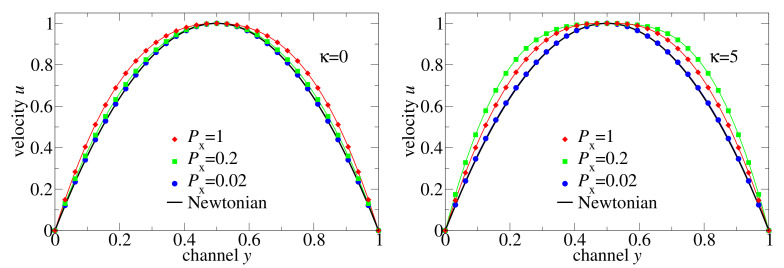
Velocity profiles of flexible and semiflexible oligomer chains in comparison with Newtonian flow profiles. The velocity profiles shown in Figure 6 were normalised by the corresponding max(u(·,y)).

To compare different non-Newtonian and Newtonian flows, we plotted in Figure 7 the velocity distributions normalised by the maximal velocity together with the Newtonian fluid solution. (i) At low-pressure drop, the flow of both flexible and semiflexible melts is Newtonian, and it deviates from the Newtonian regime as the external pressure difference increases. (ii) The non-Newtonian effect increases progressively in the flexible chain melt, but it is non-monotone for the semiflexible chains: $P_{x}=0.2$ causes a larger non-Newtonian effect on the velocity distribution than $P_{x}=1$. The reason for this retrograde non-Newtonian behaviour is the following: The semiflexible chain melt is coming into the regime of a second plateau of viscosity at high shear rates where the melt behaves like at low shear rates, but the flow velocity is larger due to lower viscosity. The conjecture on the existence of the second plateau at high shear rates is based in the existence of a positive curvature of $\eta (\dot{\gamma })$ for $\dot{\gamma }>0.01$ in Figure 5. In contrast, the microscopic data for a flexible chain does not indicate positive curvature within the measured shear rates.

**Figure 8:**
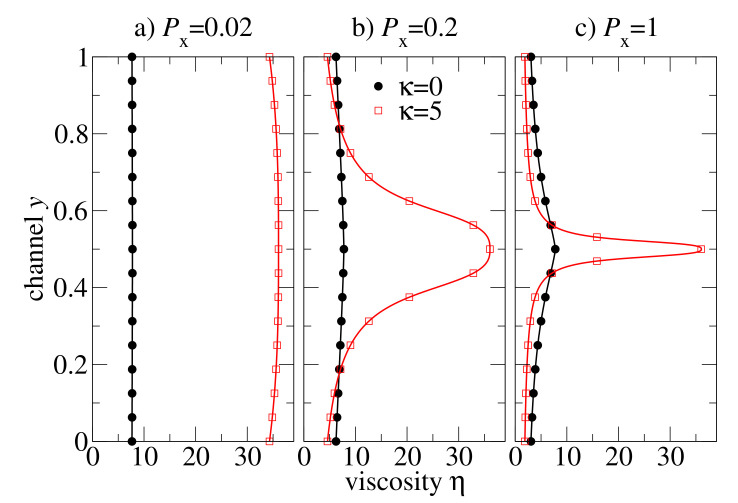
Steady-state viscosity distributions across the channel flow of flexible and semiflexible oligomer melts at various external pressure drops $P_{x}$.

We proceed with the analysis of viscosity distributions η(y), see Figure 8. For slowly flowing melts ($P_{x}=0.02$ in Figure 8a) the viscosity is almost constant in flexible and semiflexible chain melts. At moderate pressure drop $P_{x}=0.2$, the viscosity is almost constant in the flexible chain melt, but it is Λ-shaped with a large amplitude variation between the centre of the channel and near the walls for the semiflexible chain melt. At large pressure drop ($P_{x}=1$), the semiflexible chains develop a kind of viscosity spike localised at the centre of the channel due to $\dot{\gamma }\left(\ell /2\right)=0$. Except for this singular region, the viscosity distribution of semiflexible chains is very flat, and the viscosity value is smaller than in the flexible chain melt.

The second test is devoted to the study of complex geometry effects. Here we investigate non-Newtonian flows in a channel with a backward-facing step, see Armaly et al. [77]. Newtonian flows in such a channel have been studied in, e.g., [78]. Depending on the inflow velocity, this flow can be laminar, transitional or turbulent. In the laminar regime at low Reynolds number, one or more recirculation zones of the secondary flow after the expansion arise. One zone is located directly behind the backward-facing step, and it can be observed already for a very low Reynolds number. Further zones randomly appear and disappear in the case of high Reynolds numbers. In this test, there is a broad distribution of the shear rates since the intensity of the flow and the shape of streamlines are very different in the main and the secondary flows.

**Figure 9:**
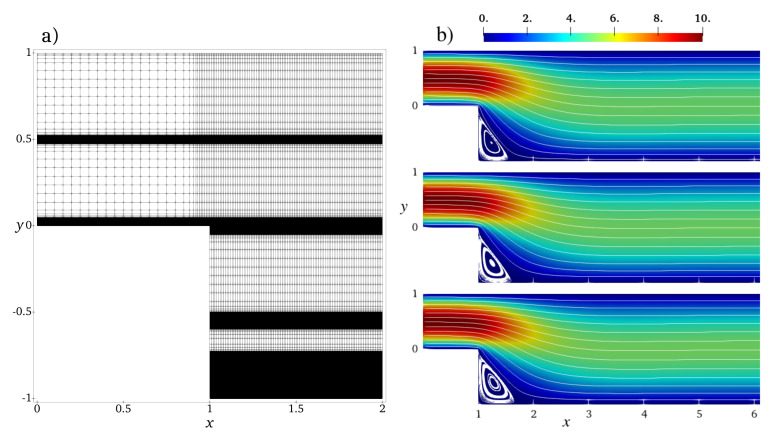
(**a**) Structured quadrilateral mesh used in the Armaly experiment: a cut–out [0…2] × [−1…1] of the full domain [0…10] × [−1…1] is shown. Mesh resolution increases smoothly at x≈1 and at y∈{−1,−0.5,0,0.5,1}. Mesh step varies between $h_{x}=1/20$ and 1/100 for the *x*–direction and between $h_{y}=1/20$ and 1/1000 for the *y*–direction. (**b**) Velocity and streamlines of the non-Newtonian flows of flexible, semiflexible chain molecules, and of the Newtonian flow, $U_{\mathrm{inlet}}=10$, κ=0 (**top**), κ=5 (**middle**), η=1 (**bottom**).

We study the flow of chain molecules in the channel of the length ℓ=10 and the height at the inlet $L_{\mathrm{inlet}}=1$ and at the outlet $L_{\mathrm{outlet}}=2$. The inlet is located at x=0. The computation domain is a structured quadrilateral mesh shown in Figure 9a. In the *x*-direction, the mesh is homogeneous with the mesh step $h_{x}=1/20$ except for the region near the backward-facing step x∈[1,2] where the grid density has been smoothly increased in order to better resolve the transit region. The fine mesh step is $h_{x}=1/100$. In the *y*-direction, the mesh has several high-resolution regions: near the walls at y∈{−1,0,1}, in the middle of the inlet part (y=0.5) and of the main part (y=0), and one zone at y=−0.5. The fine mesh resolution in these zones is $h_{y}=1/1000$, and the coarse resolution is $h_{y}=1/20$ elsewhere with a smooth transition in-between.

At the inlet at x=0, the Dirichlet boundary condition is applied for the inflow velocity $\left(u\left(0,y\right)=4U_{\mathrm{inlet}}y\left(1-y\right),v\left(0,y\right)=0\right)$, where $U_{\mathrm{inlet}}$ is the amplitude of the inflow velocity used as a control parameter for simulations. The outlet boundary conditions at x=ℓ impose zero stress (Neumann boundary conditions), i.e., σ·n=0.

**Figure 10:**
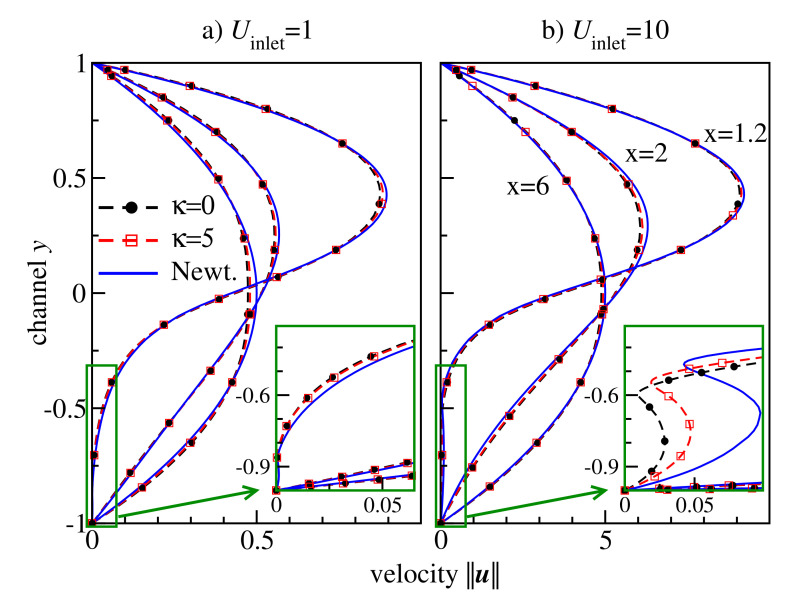
Velocity profiles at x∈{1.2,2,6} in Newtonian (blue curves) and non–Newtonian flows of flexible κ=0 (black circles) and semiflexible κ=5 (red squares) chain molecules in the Armaly experiment with $U_{\mathrm{inlet}}\in \left\{1,10\right\}$. Insets: zooming into the region of the secondary flow.

Figure 9b compares the velocity and the flow streamlines for flexible, semiflexible chain molecules and the Newtonian fluid for inflow velocity $U_{\mathrm{inlet}}=10$. Although the overall pictures look similar, one can clearly observe variations in the recirculation zone in the corner behind the step. To analyse the solution in details, we plot in Figure 10 velocity cross-sections at x=1.2,2,6 for different inflow velocities. These positions represent three characteristic regions of the flow behind the backward-facing step: the secondary flow vortex at the bottom corner directly behind the backward-facing step (x=1.2), flow directly behind the secondary flow (x=2), and flow far behind the secondary flow (x=6). The velocity profiles change between x=1.2 and x=6 from strongly asymmetric in the transit region at the backward-facing step to a symmetric one in the region far behind the step, where the characteristic non-Newtonian shape is observed. The curve for semiflexible chains is located between the curves for flexible chains and Newtonian fluid, indicating the retrograde non-Newtonian effect discussed also for the Poisseuile flow (cf. Figure 6c). This effect becomes more visible in the case of high inflow velocity, cf. the recirculation region shown in the insets of Figure 10.

**Figure 11:**
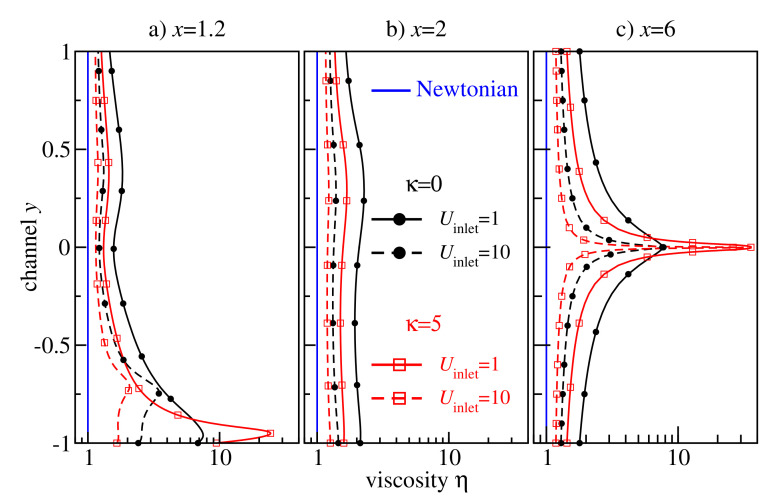
Viscosity profiles at x∈{1.2,2,6} in non–Newtonian flows of flexible (black circles) and semiflexible (red squares) chain molecules in the Armaly experiment with $U_{\mathrm{inlet}}\in \left\{1,10\right\}$. Newtonian flow viscosity η=1 is shown with blue lines.

Figure 11 shows a detailed analysis of the viscosity profiles at the cross-sections x=1.2,2,6. One sharp viscosity peak is located at the centre of the main part of the channel (y=0) far behind the backward-facing step (cf. Figure 11c). An analogous peak has already been observed for the non-Newtonian Poiseuille flow in Figure 8. Another peak arises at y<−0.5 due to the secondary flow at the corner (cf. Figure 11a), indicating that the non-Newtonian effect plays an important role in this region, too.

Our extensive simulations explain that (i) the retrograde non-Newtonian behaviour is a result of a nearly homogeneous distribution of the viscosity η(y) at high shear rates in the flow regime when $\eta (\dot{\gamma })$ does not vary significantly (post shear-thinning plateau). (ii) By tuning the flow parameters, it is possible to control the strength of the shear-thinning effect in a melt by varying the area under the Λ-shaped viscosity curve in Figure 8. (iii) A higher flow intensity leads to a wider shear rate distribution and to a narrower Λ-shaped viscosity spike. Note that the height of this spike is limited by the shear viscosity in the non-sheared system, as the flat region of the velocity profile corresponds to shear rates close to zero.

## 6. Summary and Outlook

In this manuscript, we have investigated and reviewed the molecular origins of shear-thinning at moderate to high shear rates in a high-density, low molecular weight melt of coarse-grained bead-spring polymers as a function of chain length and stiffness concisely.

In equilibrium and at low shear rates, viscosity exhibits a non-monotonous behaviour with increasing chain stiffness. While the decline (at high stiffnesses and low shear rates) has previously been associated with an isotropic-nematic transition [13], here, we attribute the initial rise with the emergence of entanglements in the melt. With increasing shear rate, this peculiar behaviour gradually vanishes as chains disentangle with the onset of collective alignment and the characteristic shear-thinning sets in. Our study thus indicates that even for rather small oligomers, rheological properties can be dominated by the presence of entanglements.

In addition, we have investigated movement modes of individual chains both in simulations of melts and single chains under shear. While in the flexible case, individual chains tumble at moderate and high shear rates [17], for semiflexible chains, we observe a transition from stretched conformations at moderate rates to a state in which stretched and tumbling chains coexist in agreement with single chain simulations in an MPCD solvent in [14]. In the near future, we plan to study explicitly the shear rate dependence of number of entanglements per chain [46,60,79,80,81,82] for the entire range of chain stiffnesses explored in this paper.

In Section 5, we have demonstrated the influence of microscopic shear-thinning behaviour on macroscopic channel flow. Using the hybrid multiscale method that couples molecular dynamics and discontinuous Galerkin schemes, we have studied flows of oligomer melts in two types of channels for flexible and semiflexible molecules, respectively. We have observed a transition from a Newtonian to a non-Newtonian flow (as well as a retrograde non-Newtonian effect for semiflexible chains) caused by the shear-thinning viscosity effects. Furthermore, we have also analysed the effects of complex geometry. In the near future, we would like to investigate shear-thinning in two-component melts consisting of polymers with varying lengths and stiffnesses as well as consequences of the latter for the macroscopic flow in simple and complex geometries. 

## Figures and Tables

**Table 1:** Reynolds numbers.

	κ=0			
		$\eta_{0}=7.76$	$\eta_{\infty }=1.084$	$\bar{\eta }=4.422$
$P_{x}$	U	$Re_{0}$	$Re_{\infty }$	Re
0.02	3.25 × 10${}^{-4}$	4.18 × 10${}^{-5}$	2.99 × 10${}^{-4}$	7.34 × 10${}^{-5}$
0.2	3.70 × 10${}^{-3}$	4.76 × 10${}^{-4}$	3.41 × 10${}^{-3}$	8.36 × 10${}^{-4}$
1	3.29 × 10${}^{-2}$	4.24 × 10${}^{-3}$	3.04 × 10${}^{-2}$	7.45 × 10${}^{-3}$
	κ=5			
		$\eta_{0}=36.05$	$\eta_{\infty }=1.093$	$\bar{\eta }=18.57$
$P_{x}$	U	$Re_{0}$	$Re_{\infty }$	Re
0.02	7.08 × 10${}^{-5}$	1.96 × 10${}^{-6}$	6.48 × 10${}^{-5}$	3.81 × 10${}^{-6}$
0.2	3.69 × 10${}^{-3}$	1.02 × 10${}^{-4}$	3.38 × 10${}^{-3}$	1.99 × 10${}^{-4}$
1	5.49 × 10${}^{-2}$	1.52 × 10${}^{-3}$	5.03 × 10${}^{-2}$	2.96 × 10${}^{-3}$

**Figure A1:**
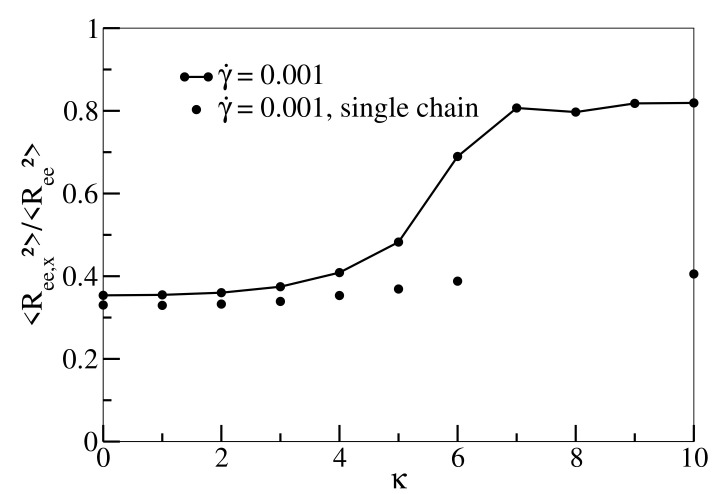
$\langle R_{ee,x}^{2}\rangle /\langle R_{ee}^{2}\rangle$ as a function of κ at $\dot{\gamma }=0.001$ for melt (line with dots) and single chain simulations (only dots) with the more involved mapping described in the appendix.

## Data Availability

Not applicable.

## Appendix A. Alternative Time-Scale Mapping

In addition to the simple time scale mapping between single chain simulations and melts described at the end of Section 2, here, we consider matching Rouse times for each value of κ. Note that while this procedure works well for κ≤6 and κ=10, we did not obtain a convincing exponential fit for the autocorrelation function of the sheared melt in the intermediate range of stiffnesses, likely due to the presence of entanglements. Excluding values for κ=7,8 and 9, the relative scaling factor ζ(κ)/ζ(κ=0) was 1.15 for κ=1, 1.29 for κ=2, 1.46 for κ=3, 1.81 for κ=4, 2.07 for κ=5, 2.58 for κ=6, and finally, 2.52 for κ=10. The results reported in Section 3, for the most part do not change qualitatively in comparison to the simplified matching.

If we apply this time-scale matching procedure to the results shown in Figure 2a,b, the single chain curves corresponding to κ=5 and 10 will be shifted to the left by factors of 2.07 and 2.52, respectively, which will not change our qualitative conclusions. Figure A1 (which corresponds to Figure 4b) shows $\langle R_{ee,x}^{2}\rangle /\langle R_{ee}^{2}\rangle$ as a function of κ at $\dot{\gamma }=0.001$. For single chain simulations, the ratio is no longer flat but rises marginally for larger values of κ. Again, this outcome does not change the qualitative conclusions described in the main text.

## Appendix B. Snapshots

Configuration (a) shows an entangled melt representing the viscosity maximum in Figure 4a. The same melt already exhibits partial alignment and disentanglement under shear, which leads to the pronounced drop in viscosity in the same figure. Configuration (c) is in a nematic phase and already aligned under equilibrium conditions.
